# Supplementary material for: Transition metal dichalcogenide metaphotonic and self-coupled polaritonic platform grown by chemical vapor deposition
Source: Nat Commun. 2022 Sep 23;13:5597. doi: 10.1038/s41467-022-33088-0 (PMC9508121; doi:10.1038/s41467-022-33088-0)
Supplement: Supplementary file 1 — Supplementary Information [file 41467_2022_33088_MOESM1_ESM.pdf]

Supplementary Information for

"Transition metal dichalcogenide metaphotonic and self-coupled polaritonic platform grown by chemical vapor deposition"

Fuhuan Shen[1,#], Zhenghe Zhang[2, 3#], Yaoqiang Zhou[1,#], Jingwen Ma[1], Kun Chen[4], Huanjun Chen[4], Shaojun Wang[2,3 *], Jianbin Xu[1,*], Zefeng Chen[1, 2,3*]

1. Department of Electronic Engineering, The Chinese University of Hong Kong, Shatin, N.T., Hong Kong SAR, P. R. China.
2. School of Optoelectronic Science and Engineering and Collaborative Innovation Center of Suzhou Nano Science and Technology, Soochow University, Suzhou 215006, China
3. Key Lab of Advanced Optical Manufacturing Technologies of Jiangsu Province & Key Lab of Modern Optical Technologies of Education Ministry of China, Soochow University, Suzhou 215006, China
4. State Key Lab of Optoelectronic Materials and Technologies, Guangdong Province Key Laboratory of Display Material and Technology, School of Electronics and Information Technology, Sun Yat-sen University, Guangzhou 510275, P. R. China

* Corresponding Authors. Email: zfchen@ee.cuhk.edu.hk (Z. F. Chen) and jbxu@ee.cuhk.edu.hk (J. B. Xu), and swang.opto@suda.edu.cn (S. Wang)

\# Contributed equally to this work.

* Corresponding Authors. Email: zfchen@ee.cuhk.edu.hk(Z. F. Chen) and jbxu@ee.cuhk.edu.hk(J. B. Xu), and swang.opto@suda.edu.cn(S. Wang)

Supplementary Figures

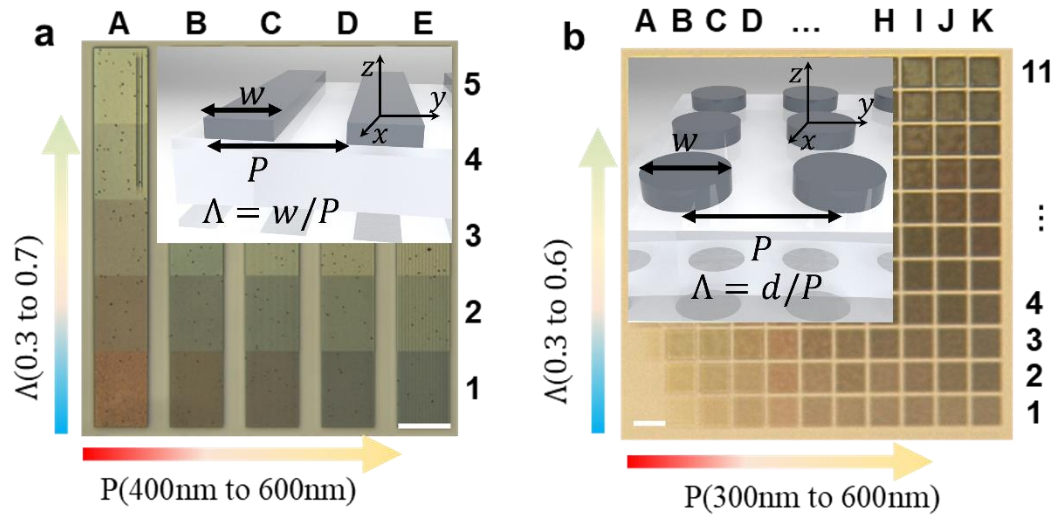

Supplementary Figure 1. Definition of geometric parameters of MoS2 metastructures. For gratings**(a)** filling factor is defined as $\Lambda = w/P$ where w is the width of the Mo grating bar(before sulfidation) and P is the period of the grating. Similar to gratings, the filling factor for disk arrays**(b)** is defined as $\Lambda = d/P$ where d is the diameter of the Mo disk. In 1D structures, A-E corresponds to the periods ranging from 400nm to 600nm with a step of 50 nm and 1-5 corresponds to the fill factors ranging from 0.3 to 0.7 with a step of 0.1. In 2D structures, A-H corresponds to the periods ranging from 300 nm to 600 nm with a step of 30 nm and 1-11 corresponds to the fill factors ranging from 0.3 to 0.6with a step of 0.03.

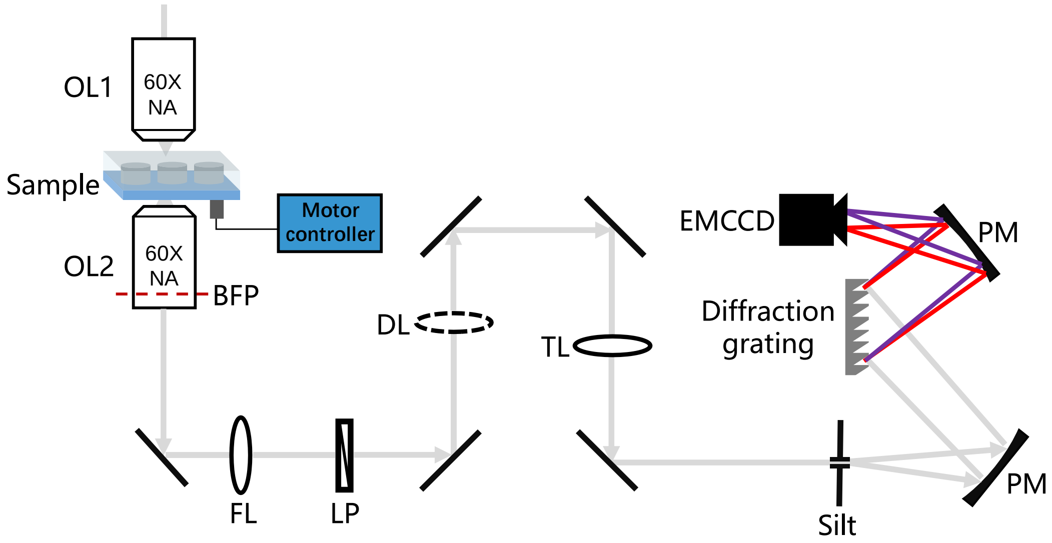

Supplementary Figure 2. Schematic Fourier imaging setup for the measurement of angle-resolved extinction spectra. OL: objective lens, FL: Fourier Lens, DL: delayed lens, TL: tube lens, LP: linear polarizer, PM: parabolic mirror, NA: numerical aperture, BFP: back focal plane.

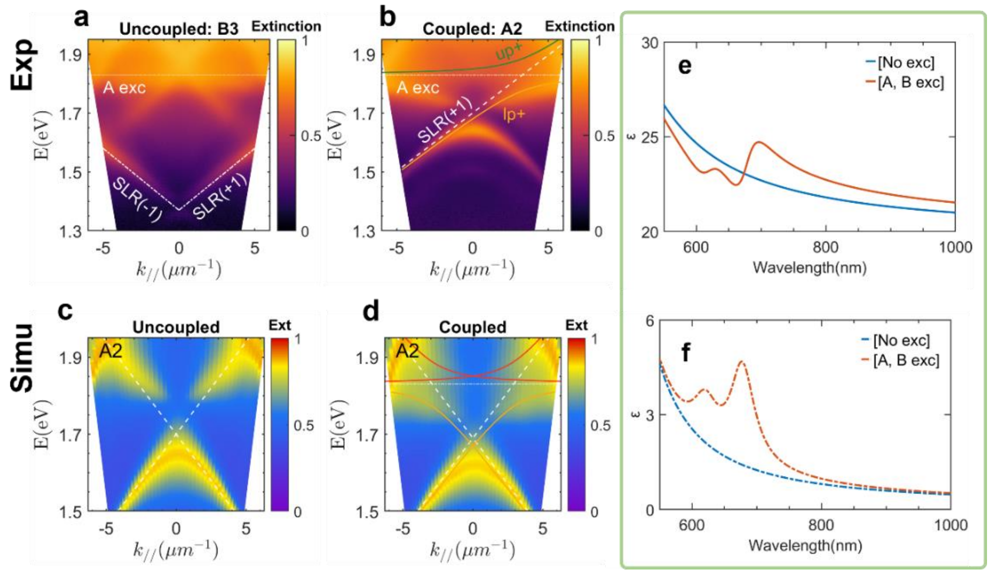

Supplementary Figure 3. (a-b) Measured angle-resolved extinction spectra of B3**(a)** and A2**(b)** samples. B3 shows the uncoupled M-SLR mode(which is linear) and A2 shows the polaritons due to the coupling of the exciton to the M-SLR mode of the 1D grating. **(c-d)** Simulated the dispersion of 1D grating for the uncoupled**(c)** and coupled**(d)** M-SLR modes with the dielectric function shown in the **(e)** and **(f)**.

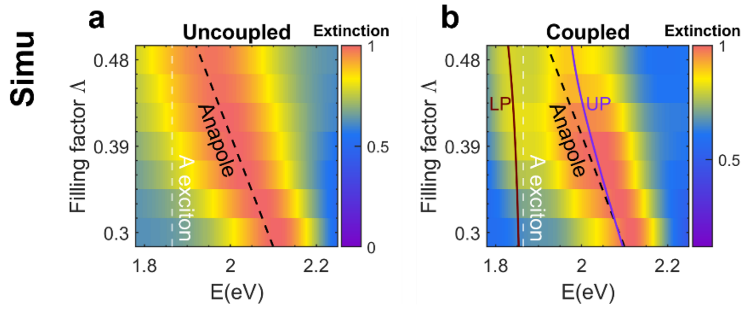

Supplementary Figure 4. Simulated extinction spectra of disk arrays without**(a)** and with**(b)** A exciton response for the uncoupled and coupled cases.The coupling strength is smaller than the linewidth of the anapole modes[1].

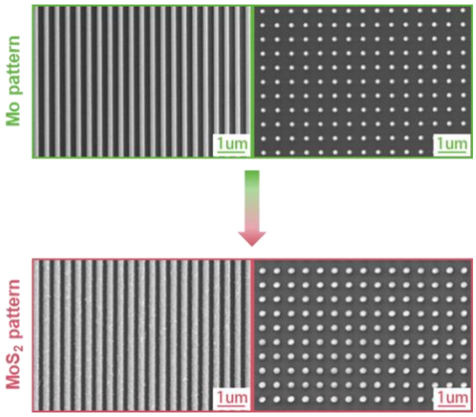

Supplementary Figure 5. SEM images of the 1D&2D Mo pattern(top) and MoS_2 pattern(bottom, after sulfidation).

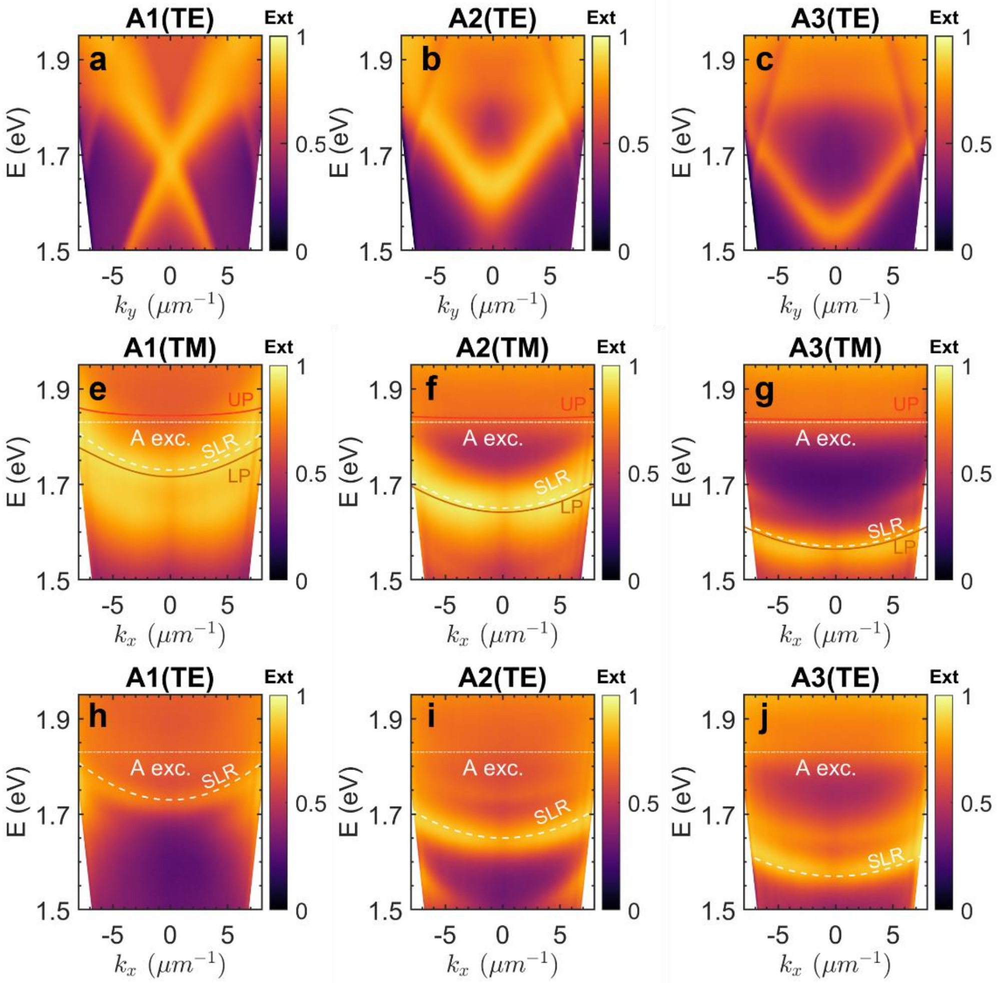

Supplementary Figure 6. (a-c) Dispersion of A1-A3 as a function of in-plane momentum k_y under TE polarized incidence. We find that for TE polarization, there is no clear anti-crossing behavior shown in the dispersions which might be ascribed to the weak interaction of exciton and electric field along the x-direction(Figure S12b). **(e-g)** Dispersion of A1-A3 as a function of in-plane momentum k_x under TM polarized incidence. In addition to the SLR(white dashed line) labeled in the **(e-g)**, there

appears the second branch shows hyperbolic dispersion which is ascribed to the k_y component(i.e., $k_y \neq 0$) during the measurements. **(h-j)** Dispersion of A1-A3 as a function of in-plane momentum k_x under TE polarized incidence. For TE modes, the coupling of photonic modes and excitons is not as ambiguous as TM modes which might be due to the unconfined fields along the yz-plane, as Supplementary Figure 6 shows.

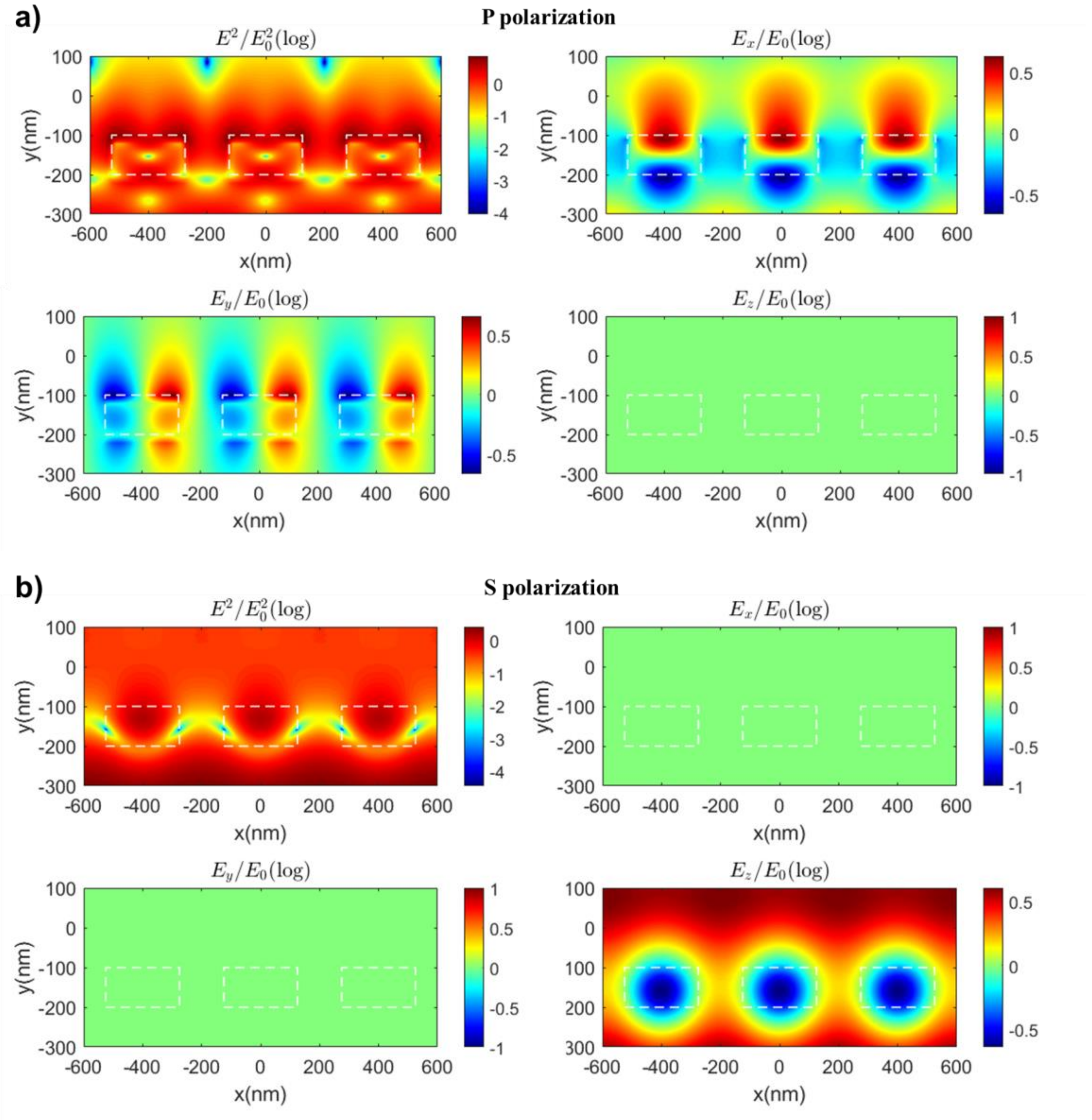

Supplementary Figure 7. (**a**) The intensity(square) as well as x-, y-, z-component of electric field distribution of the resonant mode of 1D grating under p polarization(i.e., TM polarization). They show the typical magnetic-type mode and the E_z component is zero at this mode. (**b**) The intensity(square) as well as x-, y-, z-component of electric field distributions of the resonant mode of 1D grating under s polarization(i.e., TE polarization). They show the typical electric-type mode distribution and the E_x and E_y component is zero at this mode and E_z is along the grating bar.

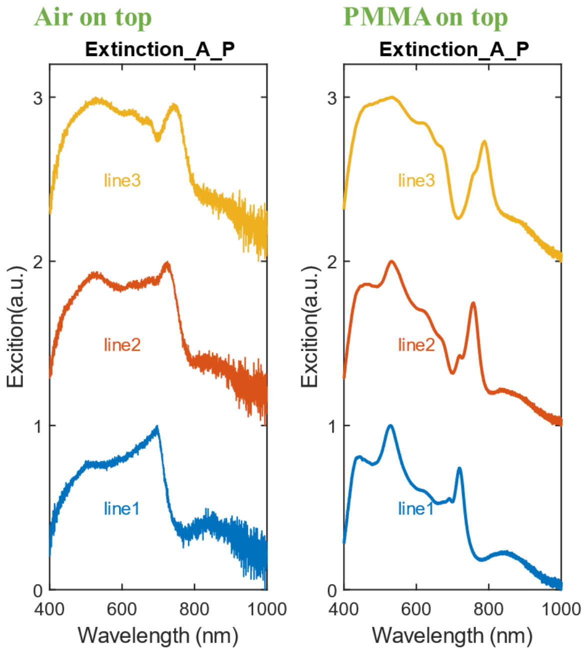

Supplementary Figure 8. Comparison of extinction spectra(p-polarization, i.e., TM polarization) of A1-A3 with air and PMMA on top. Due to the index match of bottom and top the resonant peak of 1D grating with PMMA on top is more symmetric and of reduced linewidth. As a result, we spin coat a layer of PMMA(~100nm) on the top of the MoS_2 metastructures for the optical measurements. Line1 to line 3 indicate various filling factors(as illustrated in the Supplementary Figure 1).

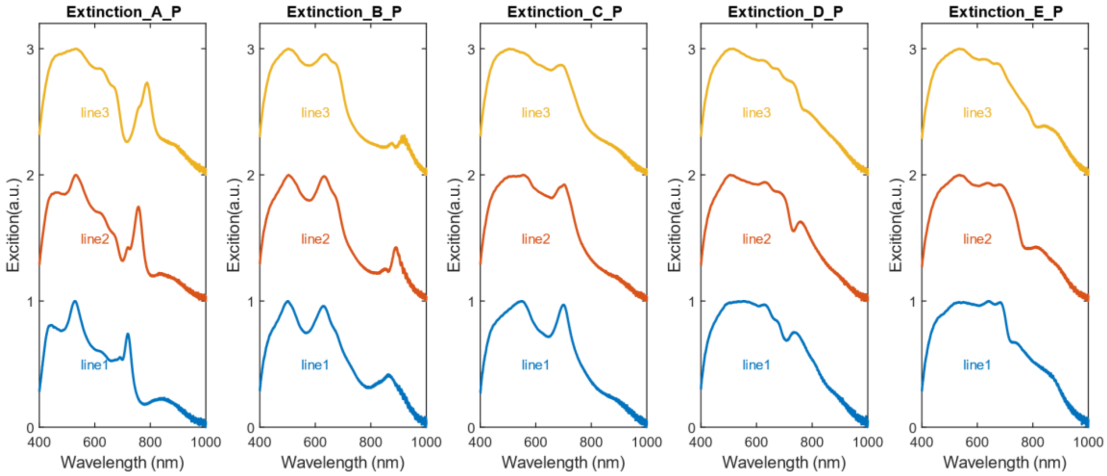

Supplementary Figure 9. Extinction spectra for A-E(400nm to 600nm) of the 1D grating under p polarization(i.e., TM modes).

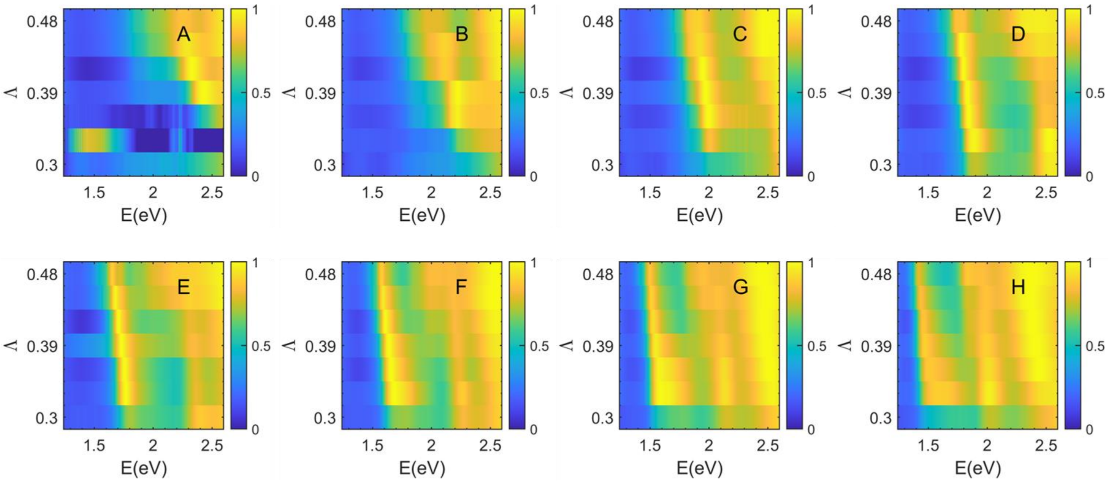

Supplementary Figure 10. The extinction spectra for 2D MoS_2 disk arrays. A-H indicates the period ranges from 300nm to 510nm.

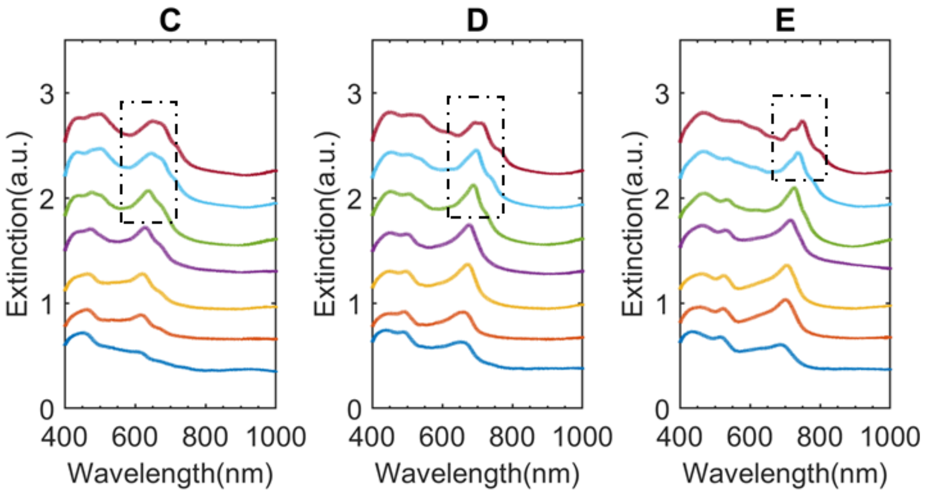

Supplementary Figure 11. Separated ED and MD modes in the extinction spectra for 2D MoS_2 structures.

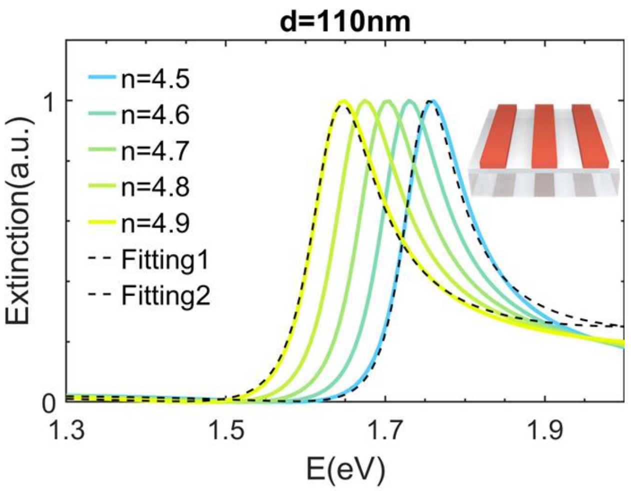

Supplementary Figure 12. Simulated extinction spectra for 1D MoS_2 metastructure as the function of the refractive index of the material(n from 4.5 to 4.9). The period is fixed as 400nm and the width of the grating bar is 110nm. The dashed curves are the fitting curves based on the Fano formula in ref[3].

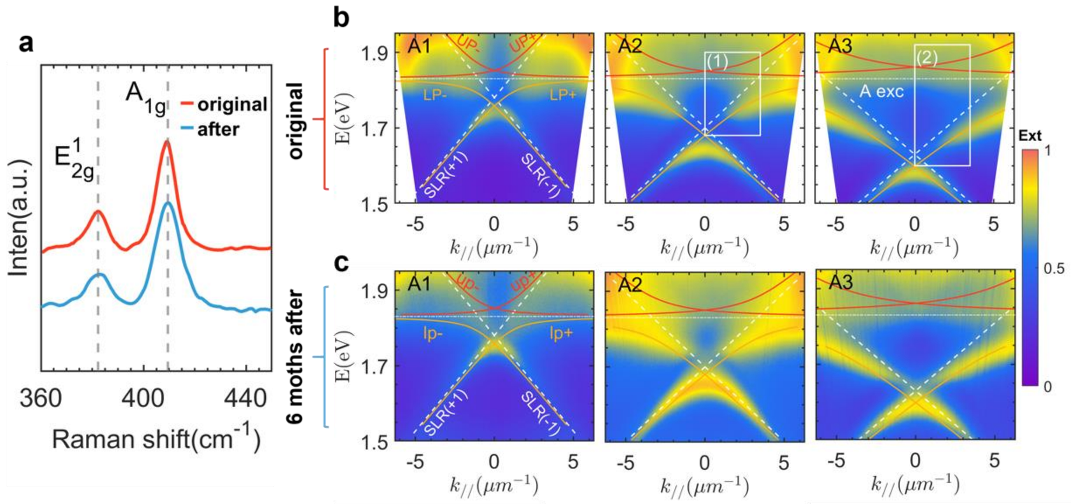

Supplementary Figure 13. (a) Original(red) Raman spectra of MoS2 nanostructure and the spectra after more than 6 moths(blue). **(b)** Dispersion of A1-A3 for original measurements(i.e., Fig. 4a in the revised manuscript) and **(c)** measurement results after more than 6 months.

Supplementary Tables

	$\hbar\omega_{cav}$(eV) (Normal incidence)	$\hbar\omega_A$(eV)	g_A(meV)
Exp(A1)	1.76	1.83	40
Exp(A2)	1.7	1.83	55
Exp(A3)	1.63	1.83	85
Simu(A1)	1.82	1.83	40
Simu(A2)	1.675	1.83	55
Simu(A3)	1.645	1.83	85

Supplementary Table 1. The parameters to fit the figures shown in Fig 4a and Fig 4b in the main text where the dispersion relation of eq(20) is applied in the fitting process.

Material	Range of κ (750nm to 1000nm)	Source
MoS_2	10^{-2} to 10^{-4}	Haonan Ling et al[4] (our work is within the range)
Ge	10^{-1}	Nunley et al[5]
Si	10^{-2} to 10^{-4}	Haonan Ling et al[4]
GaAs	10^{-1} to 10^{-5}	Papatryfonos et al[6]

Supplementary Table 2. Comparisons of extinction coefficients of MoS_2 and some other semiconductors.

Supplementary Notes

Supplementary Note 1. CQED theory to describe the system

We firstly start from a simplest case where decays of exciton and cavity are ignored, to give the ensemble coupling strength for multiple excitons coupling to the cavity. Then the decays are taken into consideration and the final optical response of the total system is calculated.

(1) Ensemble coupling strength

In the beginning, several rational assumptions in the derivation should be declared. The excitons are assumed averagely coupled with the cavity, i.e., the resonance frequency of multiple excitons is the same and so is the coupling strength. The Hamiltonian to describe the system can be given

$$H = \omega_{cav}\hat{a}^{\dagger}\hat{a} + \sum_{k} \omega_{exc}\hat{b}^{\dagger}{}_{k}\,\hat{b}_{k} + \sum_{k} g_{k}(\hat{b}^{\dagger}{}_{k}\hat{a} + h.c.) \tag{1}$$

where ω_{cav} and ω_{exc} are the resonant frequency of cavity and exciton; g_k is the coupling strength of kth exciton coupled to the cavity; $\hat{a}^{\dagger}(\hat{a})$ and $\hat{b}^{\dagger}{}_{k}(\hat{b}_{k})$ are the creation(annihilation) operator of the cavity and kth exciton. Here, h.c. represents the Hermitian conjugate and for simplicity we set $\hbar = 1$. Applying the Heisenberg equation $\frac{\partial A_{\varkappa}}{\partial t} = \frac{1}{i}[A_{\varkappa}, H]$ and utilize the commutation relation of $\hat{a}$ and $\hat{b}_k$, i.e., $[\hat{a}, \hat{a}^{\dagger}] = 1$ and $\left[\hat{b}_k, \hat{b}^{\dagger}{}_{k}\right] = 1$ we could deduce that

$$\dot{\hat{a}} = -i\omega_{cav}\hat{a} - i\sum_{k} g_k\hat{b}_k \tag{2}$$

$$\dot{\hat{b}}_k = -i\omega_{exc}\hat{b}_k - ig_k\hat{a} \tag{3}$$

Through Fourier transformation, we then obtain the relations in the frequency domain

$$-i\omega\hat{a}(\omega) = -i\omega_{cav}\hat{a}(\omega) - i\sum_{k} g_k\hat{b}_k(\omega) \tag{4}$$

$$-i\omega\hat{b}_k(\omega) = -i\omega_{exc}\hat{b}_k(\omega) - ig_k\hat{a}(\omega) \tag{5}$$

If we define the total coupling strength

$$g^2 = \sum_{k} {g_k}^2 \tag{6}$$

And the ensemble operator

$$\hat{b} = \sum_k g_k \hat{b}_k / g \tag{7}$$

Then the equations 4 and 5 can be simplified

$$-i\omega\hat{a}(\omega) = -i\omega_{cav}\hat{a}(\omega) - ig\hat{b} \tag{8}$$

$$-i\omega\hat{b}(\omega) = -i\omega_{exc}\hat{b}(\omega) - ig\hat{a}(\omega) \tag{9}$$

Eq(6-7) has a clear physical illustration. The total effect of an ensemble of excitons can be represented by the single exciton with equivalent coupling strength to the cavity. Thus, if there are N excitons coupled in the system, we can obtain

$$g^2 = \sum_k {g_k}^2 = N{g_0}^2 \tag{10}$$

where g_0 is the coupling strength of one single exciton and the total(ensemble) coupling strength can be given by $g = \sqrt{N g_0}$.

For a single exciton coupled to the cavity, coupling strength can be expressed as

$$g_0 = \sqrt{\frac{\omega}{\varepsilon_0 Re\left[\frac{d(\varepsilon\omega)}{d\omega}\right]V}}\mu_0 \tag{11}$$

where μ_0 is the transition dipole moment of the exciton and V is the mode volume. $\varepsilon_0 Re\left[\frac{d(\varepsilon\omega)}{d\omega}\right]$ appears during the calculation of the energy density of EM field, which is due to the material dispersion and becomes $\varepsilon_0\varepsilon$ when ε is constant.

From eq(5) we could obtain that

$$\hat{b}_k(\omega) = \frac{g_k\hat{a}(\omega)}{\omega - \omega_{exc}} \tag{12}$$

Then

$$\hat{a}(\omega) = \frac{\sum_k {g_k}^2\hat{a}(\omega)}{(\omega - \omega_{cav})(\omega - \omega_{exc})} \tag{13}$$

(2) Optical response of hybrid system and dispersion of the upper&lower polaritons

Next, we take the decay rates of the cavity(κ) and exciton(γ) into the system, then the eq(8-9) has the forms

$$-i\omega\hat{a}(\omega) = -i\omega_{cav}\hat{a}(\omega) - ig\hat{b} - \frac{\kappa}{2}\hat{a}(\omega) - \sqrt{\kappa}\hat{a}_{in}(\omega) \tag{14}$$

$$-i\omega\hat{b}(\omega) = -i\omega_{exc}\hat{b}(\omega) - ig\hat{a}(\omega) - \frac{\gamma}{2}\hat{b}(\omega) \tag{15}$$

From eq(15) we obtain

$$\hat{b}(\omega) = \frac{g\hat{a}(\omega)}{\omega - \omega_{exc} - i\frac{\gamma}{2}} \tag{16}$$

Then substitute eq(16) in the eq(15) we get

$$\hat{a}(\omega) = \frac{-\sqrt{\kappa}}{\frac{\kappa}{2} + i(\omega_{cav} - \omega) + \frac{ig^2}{\omega - \omega_{exc} - i\frac{\gamma}{2}}}\hat{a}_{in}(\omega) \tag{17}$$

Since $\hat{a}_{out}(\omega) - \hat{a}_{in}(\omega) = \sqrt{\kappa}\hat{a}(\omega)$, we can deduce that

$$\hat{a}_{out}(\omega) = A(\omega)\hat{a}_{in}(\omega) \tag{18}$$

where

$$A(\omega) = 1 + \frac{-\kappa}{\frac{\kappa}{2} + i(\omega_{cav} - \omega) + \frac{ig^2}{\omega - \omega_{exc} - i\frac{\gamma}{2}}} \tag{19}$$

is called **response function** of the system.

The corresponding dispersion of the upper polariton(UP) and lower polariton(LP) can thus be deduced. To obtain the eigen energy(i.e., the loss is neglected), we have

$$\omega_{\pm} = \frac{\omega_{cav} + \omega_{exc}}{2} \pm \sqrt{g^2 + \frac{\delta^2}{4}} \tag{20}$$

where $\delta = \omega_{cav} - \omega_{exc}$. Eq(20) is the **polaritons dispersion**("+" for up and "–" for lp) in the main text.

(3) Condition to reach the strong coupling regime

The Rabi splitting(energy difference between UP and LP at $\delta = 0$) $\Omega = 2g$. In order to reach the strong coupling regime, It requires that

$$g \geq \frac{\kappa + \gamma}{4} \tag{21}$$

Supplementary Figure 14 shows the spectra based on the eq(19) with different coupling strength g. With $\kappa = 100meV$ and $\gamma = 50meV$, g should be larger than $37.5meV$ to be able to observe the Rabi splitting.

In Figure 4(a,b), the coupling strength $g_A = 85meV$, the decay rate of M-SLR $\kappa = 100meV$(not the extinction coefficient in the main text) and decay rate of A exciton

$\gamma = 140meV$. As a result, the strong coupling is realized as $g_A > \frac{\kappa+\gamma}{4}$.

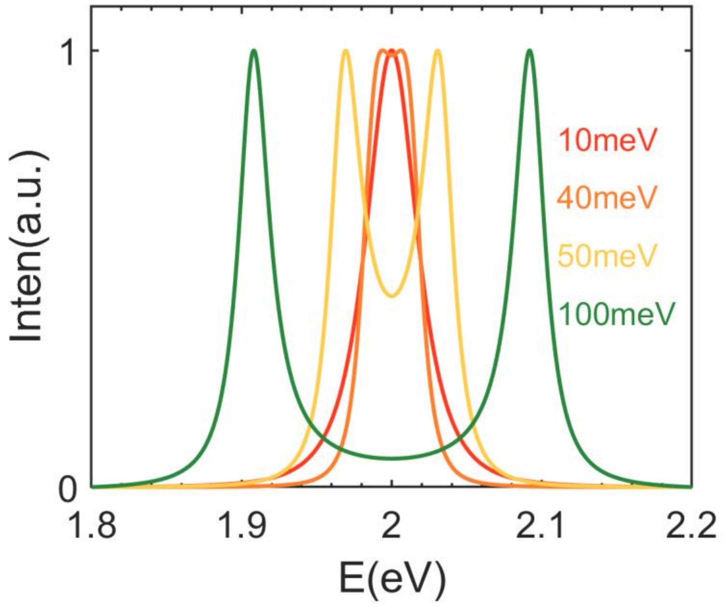

Supplementary Figure 14. Spectra of the exciton-cavity hybrid system with different coupling strengths $\boldsymbol{g}$(red: 10meV, orange: 40meV, yellow: 50meV, green: 100meV).

Supplementary Note 2. Dispersion of 1D gratings

Rayleigh's anomaly(RA) occurs when the diffraction waves propagate at the interface of the photonic crystal or grating. At this condition, spectra experience abrupt change and usually resonance peaks with a high Q factor would be generated. For instance, the high Q response shown in Fig. 2(e) is the result of the RA's anomaly due to the collective response of the elements constituting the grating. The incident wavevector k_{in} and diffraction wavevector k_d should meet the grating equation

$$\pm k_{d\parallel} = k_{in\parallel} + G \qquad (22)$$

$k_{in\parallel} = \frac{2\pi}{\lambda}\sin\theta_{in}$ and $k_{d\parallel} = \frac{2\pi}{\lambda}n_{eff}$ are the in-plane component of incident and diffraction wavevector and $G = \frac{2\pi}{P}$ is the reciprocal vector of the 1D grating. λ, n_{eff}, P and θ_{in} are the wavelength in the free space, effective refractive index, period of the 1D grating, and incident angle of the illumination respectively. Here, for simplicity, we use a scalar equation instead of a vector equation for the 1D condition.

In this work, we focus on the dispersion of the 1D grating structure. As Supplementary Figure 15(also shown in Figure 3a in the main text) shows, the reciprocal vector of the grating is along the y-direction $G = \frac{2\pi}{P_y}$. The dispersion relation is dependent on the incidence direction(i.e., θ_x and θ_y). For k_y, the energy-

momentum dispersion(as Supplementary Figure 15b shows) can be described by

$$E(\pm 1) = \mp \frac{\hbar c}{n_{eff}} (k_y \pm G) \tag{23}$$

where the resonant energy is linearly related to the in-plane component k_y. For k_x, the energy-momentum dispersion(as Supplementary Figure 15c shows) can be described by

$$E(\pm 1) = \mp \frac{\hbar c}{n_{eff}} \sqrt{{k_x}^2 + G^2} \tag{24}$$

which is the typical parabolic dispersion. The negative branch can be ignored.

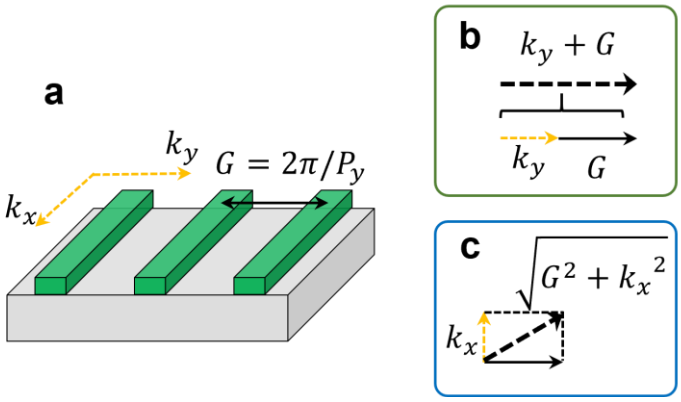

Supplementary Figure 15. (a) Schematic illustration of 1D grating with the definition of reciprocal vector G and the in-plane component of incident wavevector k_x and k_y.

Supplementary Note 3. Measuring the refractive index of MoS_2

Following the work by Yilei Li et al[2], we combined the multiple-Lorentz model and the transfer matrix method to fit the transmission(extinction) spectrum we obtained in the experiment. The dielectric function of the bulk MoS_2 can be described by the multiple-Lorentz model:

$$\varepsilon_{MoS2} = \varepsilon_0 + \sum_{i=1}^{3} f_i \frac{{\omega_i}^2}{{\omega_i}^2 - \omega^2 - j\gamma_i \omega} \tag{25}$$

where $i = 1, 2, 3$ represent responses of the A, B, and C excitons.

To obtain the refractive index of the bulk MoS_2 after CVD process, we first measure the extinction spectra of MoS_2 film. The experiment setup, AFM map, and height profile are shown in Supplementary Figure 16(a-c). Then the transfer matrix method(TMM) is applied to calculate the extinction of the MoS_2 film. Compared to the refractive index shown in the ref[2], the oscillator strength for A exciton is slightly weaker than the exfoliated counterpart which is due to the strain and defected after the high-temperature CVD process. <u>The refractive index of MoS_2 might be slightly modified(within 20%) to show the best fit of the simulated results to the experiment measurements.</u>

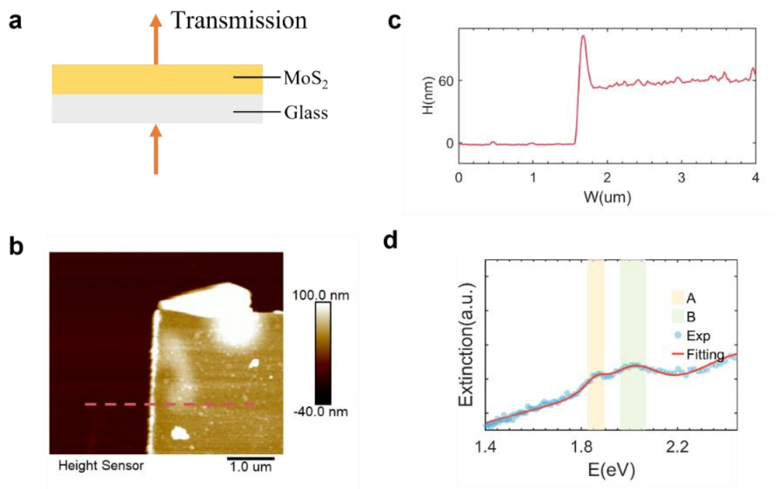

Supplementary Figure 16. **(a)** A schematic figure for the experiment and TMM calculation setup. **(b)** AFM figure of the MoS_2 film. **(c)** The cross-section of the red dashed line indicated in **(b)**. **(d)** Measured extinction spectra(blue circles) of the MoS_2 shown in **(b)** and fitting curves(red line) calculated from the TMM.

Supplementary Note 4. FDTD simulations

A finite-difference time-domain (FDTD) solver from Lumerical Inc. is used. The refractive index of MoS_2 is inserted in the material lab of the software, whose value may be slightly adjusted to get the best fitting to the experimental results.

The simulations for 1D MoS_2 grating were performed by the 2D FDTD simulation region using the periodic boundary condition in the y-axis and perfectly matched layer(PML) in the x-axis(as schematically shown in Supplementary Figure 17(a)). The illumination consists of a broadband (400–1000 nm) plane wave beam(bloch/period type), which is incident normal to the substrate. Power transmission monitors were placed at the back side of the substrate to detect the transmission spectra. Mesh sizes for both x and y axes were set as 5nm.

The simulations for 2D MoS_2 disk array were performed by 3D FDTD simulation region using the periodic boundary condition in the x- and y-axes and perfectly matched layer(PML) in the z-axis(as schematically shown in Supplementary Figure 17(b)). The plane wave(bloch/period type) is along the z-axis for normal incidence. Power transmission monitors were placed at the back of the substrate to detect the transmission spectra. Mesh sizes for both x- and y- and z-directions were set as 5nm.

For the angle-resolved spectra(Fig 4b, 4e), the type of plane wave was selected as BFAST. The bloch boundary condition was chosen in the y direction and PML for the x direction. By varying the incident angle(as the orange dashed arrow in Figure S6(a) shows) from 0 degrees to 40 degrees (with a step of 2 degrees), we obtained the spectra with different incident angles (i.e., in-plane momentum k_y).

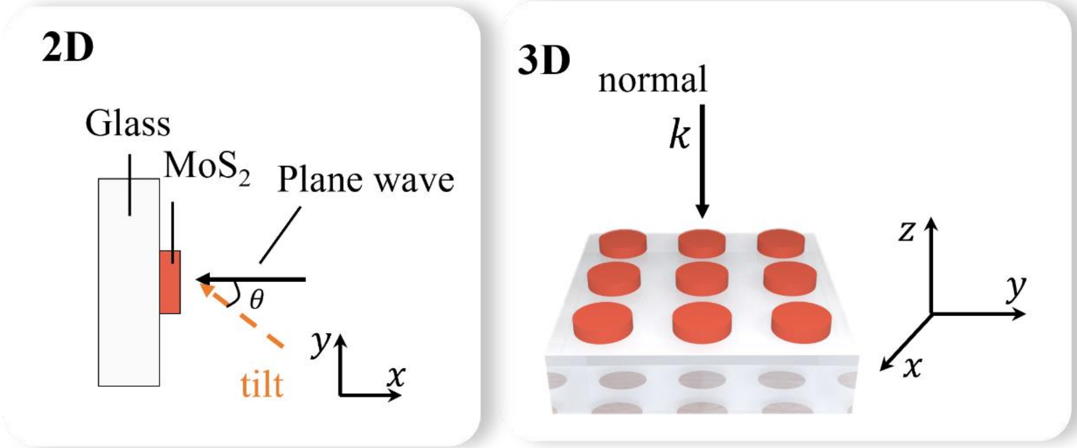

Supplementary Figure 17. Schematic setup for 2D**(a)** and 3D**(a)** FDTD simulation region.

To calculate the mode area we insert the *mode_area analysis group*(contained in the FDTD software) at the cross section(yz-plane in Figure S7). We select the resonance frequencies(i.e., the M-SLR mode) for A1-A3 and calculate the corresponding mode areas respectively. Supplementary Figure 18 shows the $|E/E_0|^2$ distributions for A1-A3 which are calculated by the power monitor in FDTD software, indicating different field distributions in space for A1-A3.

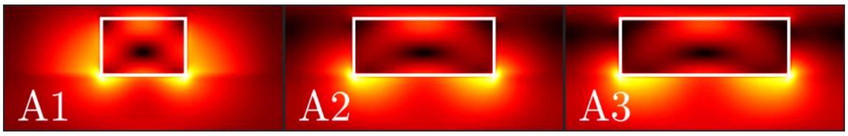

Supplementary Figure 18. $|E/E_0|^2$ distributions for A1, A2 and A3 at the resonant peaks.

Supplementary References:

[1] Wang, H. et al. Resonance Coupling in Heterostructures Composed of Silicon Nanosphere and Monolayer WS2: A Magnetic-Dipole-Mediated Energy Transfer Process. ACS Nano **13**, 1739–1750 (2019).

[2] Li, Y. et al. Measurement of the optical dielectric function of monolayer transition-metal dichalcogenides: MoS2, Mo S e2, WS2, and WS e2. Phys. Rev. B - Condens. Matter Mater. Phys. **90**, 1–6 (2014).

[3] Wang, M. et al. Tunable Fano Resonance and Plasmon–Exciton Coupling in Single Au Nanotriangles on Monolayer WS2 at Room Temperature. Adv. Mater. **30**, (2018).

[4] Ling, H., Li, R. & Davoyan, A. R. All van der Waals Integrated Nanophotonics with Bulk Transition Metal Dichalcogenides. ACS Photonics **8**, 721-730 (2021).

[5] Nunley, T. N. et al. Optical constants of germanium and thermally grown germanium dioxide from 0.5 to 6.6eV via a multisample ellipsometry investigation. J. Vac. Sci. Technol. B, Nanotechnol. Microelectron. Mater. Process. Meas. Phenom. **34**, 061205 (2016).

[6] Papatryfonos, K. et al. Refractive indices of MBE-grown AlxGa(1- x)As ternary alloys in the transparent wavelength region. AIP Adv. **11**, (2021).
